# Aspergillus and Fusarium Mycotoxin Contamination in Maize (*Zea mays* L.): The Interplay of Nitrogen Fertilization and Hybrids Selection

**DOI:** 10.3390/toxins16070318

**Published:** 2024-07-13

**Authors:** Muhoja Sylivester Nyandi, Péter Pepó

**Affiliations:** 1 Kálmán Kerpely Doctoral School of Crop Production and Horticultural Science, University of Debrecen, Böszörményi Street 138, H-4032 Debrecen, Hungary; 2 Department of Crop Science and Beekeeping Technology, College of Agriculture and Food Technology, University of Dar es Salaam, P.O. Box 35134, Dar es Salaam 14115, Tanzania; 3 Institute of Crop Sciences, Faculty of Agricultural, Food Sciences and Environmental Management, University of Debrecen, Böszörményi Street 138, H-4032 Debrecen, Hungary

**Keywords:** maize, hybrid, nitrogen fertilization, mycotoxins, *Fusarium graminearum*, *Fusarium verticillioides*, *Aspergillus flavus*

## Abstract

Maize plays a significant global role as a food source, feed, and as a raw material in industry. However, it is affected by toxin-producing fungi, mainly *Fusarium graminearum*, *Fusarium verticillioides*, and *Aspergillus flavus*, which compromise its quality. This study, conducted in 2022 and 2023 at the Látókép long-term research site of the University of Debrecen, Hungary, investigated the effects of different nitrogen fertilization rates (0, 90 and 150 Kgha^−1^ N) on mycotoxin contamination (DON vs. FB vs. AFB1) in the kernels of three (3) maize hybrids: DKC4590 (tolerant), GKT376 (sensitive), and P9610 (undefined). The results showed a significant (*p* = 0.05) influence of nitrogen fertilization and maize genotype on mycotoxin levels. Sole nitrogen impacts were complex and did not define a clear trend, contrary to the hybrids selected, which followed superiority to resistance. Increased nitrogen fertilization was associated with higher DON production, while hybrid selection demonstrated a clearer trend in resistance to mycotoxins. Therefore, to maximize yield and minimize mycotoxin contamination, future research should focus on optimizing nitrogen application rates and breeding for resistance to balance yield and mycotoxin management. These results suggest that while nitrogen fertilization is crucial for maximizing yield, selecting less susceptible maize hybrids remains vital for minimizing mycotoxin contamination.

## 1. Introduction

Maize (*Zea mays* L.) contributes significantly to global agri-food systems as food, feed, and as a raw material in industry. Over the past decade, maize production has exceeded one billion metric tons annually, surpassing rice and wheat [1], driven by increased demand, technological advancements, area expansion, and yield improvements [2]. It is a significant staple and provides approximately 20% of food calories in many countries, especially in sub-Saharan Africa (SSA), Latin America, and parts of Asia [3]. It is expected to be the most traded cereal in the next few decades [2]. 

However, maize yield and grain quality is significantly compromised by mycotoxin contamination from toxigenic fungi, such as *Aspergillus flavus*, *Fusarium verticillioides*, and *Fusarium graminearum*, which produce three main mycotoxins, namely aflatoxins, fumonisin and deoxynivalenol (DON), respectively, among others [4]. These mycotoxins, occurring in agricultural commodities before or after harvest [5], pose significant health risks to humans and animals, being mutagenic, carcinogenic, teratogenic, estrogenic, hemorrhagic, immunosuppressive, nephrotoxic, hepatotoxic, and cytotoxic [6]. However, the risk of contamination is influenced by environmental conditions, insect damage, fungal infection during critical developmental stages, and nitrogen management [7,8,9]. 

Nitrogen is essential for maize growth, affecting yield, quality, and susceptibility to mycotoxin contamination [10]. However, the relationship between nitrogen fertilization and mycotoxin levels is complex, with studies reporting varying effects. In agricultural systems, maize requires nitrogen from external sources, such as organic matter, minerals, or commercial fertilizers [11]. Without supplemental nitrogen, its productivity would decline [12]. As an essential nutrient, nitrogen affects numerous parameters related to the growth and development of the plant, which directly or indirectly influence crop yield and quality [13]. It has been shown to impact the protein and oil content of maize [14], the balance between protein and starch [15], and the extent of mycotoxin contamination and fungal infection [16]. It is widely recognized that nitrogen availability influences maize susceptibility to mycotoxin contamination. The relationship between nitrogen fertilization and mycotoxin contamination is complex; there are reports of increased, decreased, and unaffected toxin contamination in response to added nitrogen [9,13,17]. These inconsistent results suggest that other interacting factors, such as hybrid genetics and environmental conditions, maybe more critical in determining mycotoxin accumulation in maize with varying nitrogen levels. However, most studies have been conducted at relatively high nitrogen levels, highlighting the need to quantify the effects of nitrogen fertilization across a broader range of nitrogen inputs. Understanding these dynamics across different nitrogen levels and maize hybrid genetics is crucial to develop effective management to reduce the risk of mycotoxin exposure to humans and animals.

Hybrid selection could play a significant role in minimizing the extent of mycotoxin contamination. It has been confirmed that resistant hybrids show reduced susceptibility to fungal infections, even though there is no complete resistance [18]. Identifying hybrids with both high yield potential and mycotoxin resistance is critical for sustainable maize production, and finding resistant maize genotypes has been a primary strategy to combat mycotoxin issues [19], with research on fungus-resistant germplasm being a global focus [20,21,22,23,24,25,26,27,28]. However, these hybrids often exhibit reduced susceptibility rather than complete resistance due to the complex selection of multiple traits and the associated genes that contribute collectively to plant resistance. Commercial maize hybrids vary in their sensitivity to infection by toxin-producing fungi, and tolerance information is rarely disclosed to users [3]. The screening of commercial hybrids has shown that tolerance levels can significantly reduce disease severity and subsequent mycotoxin production [4,29,30]. Evidence suggests that some maize hybrids are more resistant to infection by fumonisin-producing fungi and can maintain kernel integrity with less fumonisin accumulation [31]. For DON production, most studies have indicated that hybrids that are more resistant to Gibberella ear rot have lower levels of DON in infected grain [32] and aflatoxin [33]. Despite these findings, hybrids that are resistant to fungi and mycotoxins may have trade-offs in yield potential or other desirable attributes. This necessitates a balanced approach in selecting hybrids that exhibit resistance to mycotoxins and maintain high agronomic performance.

Despite extensive research, how nitrogen affects mycotoxin contamination under varying environmental conditions and in different maize hybrids remains poorly understood. A comprehensive understanding of the complex interactions between nitrogen fertilization and mycotoxin contamination could identify specific scenarios where high grain yields offset the high contamination risks. This would enable growers to achieve their agronomic goals with minimal economic and health costs. It is crucial to identify hybrids that exhibit both good mycotoxin resistance and overall agronomic performance. Given the significance of hybrid selection and nitrogen management in reducing mycotoxin contamination, this study was initiated to evaluate the effects of nitrogen fertilization on a range of pre-defined hybrids on the mycotoxin contamination of maize.

## 2. Results

In 2022 and 2023, the effects of the treatments were significant and influenced ear rot severity and mycotoxin contamination, as shown in Table 1.

### 2.1. Nitrogen Fertilization, Hybrids Tolerance, Ear Rot Severity, and Mycotoxin Contamination

The impact of nitrogen fertilization and hybrid tolerance on ear rot severity and mycotoxin production was evaluated (Table 1). Nitrogen fertilization treatments significantly (*p* < 0.05) influenced ear rot severity for FV % and FB production by 200 and 121% in 2022 between N0 and the other doses (N90 and N150), while FB production was significantly reduced with N fertilization in 2023. N fertilization was insignificant for the ear rot severity of FG%, as well as the DON toxins produced. On the other hand, hybrid tolerance was significant (*p* < 0.05) for FV% and DON production in 2022 and FG% and FB production in 2023. The influence depended on the tolerance level of the hybrids following hybrid superiority. Despite AF% being significant (*p* < 0.05) in all years, AFB1 production was only significant (*p* < 0.05) in 2022, the only year in which it was recorded. Inoculation treatments significantly affected % ear rot severity and produced mycotoxins across all nitrogen rates and hybrid tolerances in all years. 

### 2.2. Interactions between Nitrogen Fertilization and Hybrids on Ear Rot Severity and Mycotoxin Contamination 

#### 2.2.1. Influence of Nitrogen Fertilization and Hybrids on Ear Rot Severity

The treatment combinations significantly impacted ear rot severity and mycotoxin contamination in 2022 and 2023, as presented in Table 2. In artificially inoculated plots, the treatment influence on ear rot severity was dynamic and significantly (*p* < 0.05) varied from 9.6–40.1%; 0.09–0.66% and 0.04–0.77% for FG, FV, and AF in 2022, and from 22.9–67.3%; 0.23–0.56% and 0.2–0.4% for FG; FV, and AF in 2023. In treated plots, the percentage (%) of ear rot severity for FG was the most elevated, and higher by 51%, from 2023 to 2022. However, the ear rot severity in non-inoculated controls was insignificant across fertilization and selected hybrids in the two years. In all years, the less susceptible hybrid DKC4590 recorded the lowest FG% ear rot severity in all fertilizer rates compared to other hybrids.

#### 2.2.2. Effects of Nitrogen Fertilization and Hybrids on Mycotoxin Contamination

The interactive effect of nitrogen fertilizer application rate and hybrid genotypes on the amount of mycotoxin contamination evaluated is shown in Table 3. Regarding mycotoxin contamination, aflatoxin contamination was significant (*p* < 0.05) in 2022 across fertilizers rates and hybrids; however, the detected amount was below the allowed limits of contaminations for animal feeds (20 ppm) and foods (4 ppm) [34,35,36]. The addition of nitrogen increased contamination by 89% from the highest detected amount of 0.129 ppm for GKT376 at N150, and the lowest amount of 0.014 ppm for P9610 at N0 in 2022, while no aflatoxin was detected in inoculated plots and non-inoculated plots in 2023. 

The fusarium mycotoxins, DON and FB, significantly differed (*p* < 0.05) between all nitrogen rates and hybrids used in the two years. DON contamination in artificially inoculated plots followed the same trend in % ear rot severity for FG, which was higher in 2023 than in 2022. In all years, the hybrid DKC4590 significantly (*p* < 0.05) recorded the lowest amount of DON than other hybrids, following the trend DKC < GKT376 < P9610 in 2022 and DKC < P9610 < GKT376 in 2023. In uninoculated controls, DON production was insignificant and detected only for the hybrid GKT376 at N150 in 2022, while there was no detection in all hybrids and N rates in 2023. On the other hand, FB toxin production in inoculated maize significantly differed (*p* < 0.05) between hybrids in all N rates in all years. However, within N rates, it was significant (*p* < 0.05) at N150 in 2022 and N90 and N150 in 2023. In all years, the highest FB amount was recorded for the hybrid GKT376 (6.59 ppm at N150 in 2022 and 8.8 ppm at N90 in 2023), while the lowest record was DKC4590 across N rates in the two years. In non-inoculated controls, 2022 showed significant (*p* < 0.05) FB values between hybrids at N150; the detected amount was insignificant in 2023 in all hybrids across all N rates. 

### 2.3. Relationship between Ear Rot and Kernel Severity and Production of Mycotoxins

**Figure 1:**
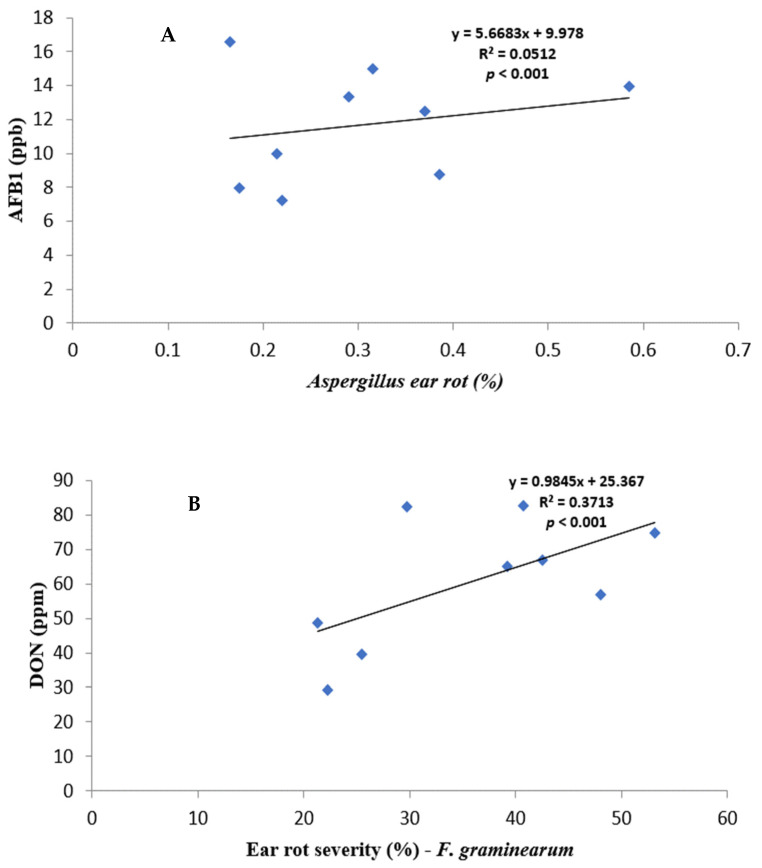

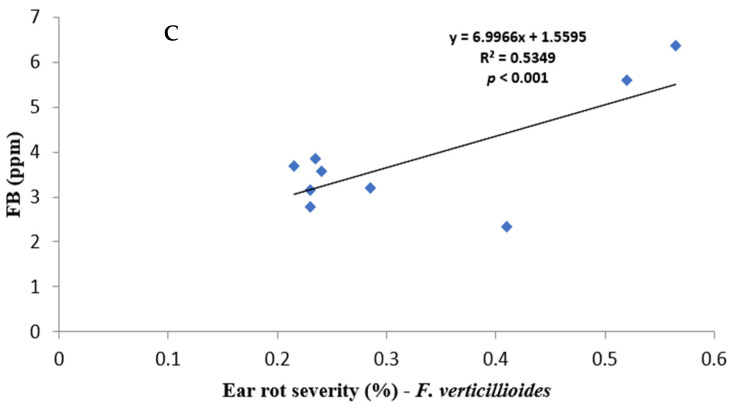
Regression relationship between ear rot severity and mycotoxin contamination: (**A**) AF% vs. AFB1; (**B**) FG% vs. DON, and (**C**) FV% vs. FB.

Regression and correlation analyses were performed to determine how much mycotoxin is produced as a result of the effect of the nitrogen added on selected hybrids to ear rot severity (Figure 1 and Table 4). We used the pooled general means to perform a regression analysis to study the relationship between ear rot severity within fungal species, and a two-tailed Pearson correlation to study the same between fungal isolates. 

All fungal species showed a positive relationship with varying strengths between ear rot severity and respective mycotoxin production. AF% ear rot severity, and AFB1 production, showed a relatively weak correlation of r = 0.2264, but it was significant (*p* < 0.001). FV% ear rot severity and total FB production indicated a strong correlation of r = 0.7313496 and it was significant (*p* < 0.001). FG% ear rot severity significantly (*p* < 0.001) correlated—r = 0.6093—to the production of DON. A relatively weak negative correlation was recorded between FG% ear rot severity and AFB and FB contamination. In contrast, AF% ear rot severity and FUM production, as well as FV% ear rot severity and AFB1 production, showed positive significant correlations. 

## 3. Discussion

The discussion section is divided into three parts. The first and second parts are dedicated to the influence of nitrogen fertilization and hybrids on the mycotoxin contamination of maize; the third part is about the interactions between the two discussed factors and the relationship between fungal ear rot severity and mycotoxin contamination. Studies show the complexity and inconsistency of how the individual nitrogen applied affects fungal ear rot severity and the resulting production of mycotoxin. At the same time, studies indicate a positive relationship between hybrid tolerance and mycotoxin contamination. Nitrogen application is essential to maximizing yield; fertilizing with a suitable hybrid in agronomic traits and tolerance would help quantify the amount of toxin produced in added nitrogen.

### 3.1. Effects of Nitrogen Fertilization on Mycotoxin Contamination

Nitrogen fertilization plays a significant role in affecting both mycotoxin contamination and maize yield. The observed inconsistencies in mycotoxin levels with varying nitrogen application rates in this study highlight the complexity of this relationship. While some reports indicate an increase in mycotoxin contamination with nitrogen application rate, others report contradictory research results. These disparities highlight the need for a comprehensive understanding of the interaction between nitrogen application, ear rot severity, and mycotoxin production. Our study revealed a general increase in mycotoxin contamination with nitrogen fertilization for AFB1 and FB in 2022. This contradicts the findings by Blandino et al. [15], who reported increased fumonisin contamination in maize due to nitrogen deficiencies.

Similarly, Tubajika et al. [37] reported reduced aflatoxin contamination by 34–45% when 50–250 KgN/ha was applied compared to N0 plots. However, the dynamics shifted in 2023, with no AFB1 recorded, while DON toxin contamination decreased with increasing nitrogen rates when used alone, resulting in the highest DON contamination at N0 in all years (Table 1). These inconsistencies align with previous studies, emphasizing the intricate relationship between nitrogen fertilization, ear rot severity and mycotoxin contamination in maize production [38,39,40,41]. The susceptibility of maize to mycotoxin contamination is dynamically influenced by nitrogen fertilization, with evidence suggesting that applied nitrogen increases and decreases mycotoxin contamination [9,17]. It appears that other interacting factors, such as hybrid genetics and environmental conditions, may be more critical in determining how much mycotoxin accumulates in maize in response to additional nitrogen, given the erratic effects of nitrogen fertilization on mycotoxin contamination. Quantifying the impacts across a range of nitrogen inputs is crucial, as most research has been conducted at relatively high nitrogen levels. This approach could provide a more comprehensive understanding of the complex interactions between nitrogen fertilization and mycotoxin contamination, facilitating the development of targeted agronomic management approaches to reduce mycotoxin risks in maize production.

### 3.2. Influence of Hybrids on Mycotoxin Contamination

The results revealed a significant effect of hybrid selection on mycotoxin contamination in all years. Consistent with findings by Bocianowski et al. [42], maize hybrids exhibiting tolerance superiority showed reduced levels of mycotoxin contamination. The less susceptible hybrid DKC 4590 consistently exhibited the lowest mycotoxin levels, whereas GKT376 demonstrated elevated mycotoxin levels. The hybrid P9610, with unknown tolerance but higher yields, was not significantly different to GKT376 in most cases for all toxins, except it recorded the lowest and most significant FB than all the others in 2023. With this exception, the overall results agree with other studies, suggesting that hybrids with good tolerance reduce the mycotoxin contamination risks [4,29,30]. Since the pre-defined hybrids have known resistance, this study does not align with Barošević et al. [43], which observed inconsistencies between hybrids in mycotoxin contamination in maize due to unknown resistances. This difference could be attributed to the known resistance characteristics of the maize hybrids used in our study, highlighting the importance of selecting hybrids with documented resistance traits to reduce mycotoxin and associated risks. A key insight of this study is that hybrid susceptibility to mycotoxin contamination depended on the specific mycotoxin and hybrid feature [44]. The different hybrids greatly influenced all the fungi: *A. flavus*, *F. verticillioides*, and *F. graminearum* mycotoxin. These results universally imply that the diverse responses of maize hybrids to different mycotoxins highlight the complexity of the interaction between the genetic landscape of the hybrids used and mycotoxin production. Overall, the different responses of the hybrids to the various mycotoxins and the potential trade-offs between increased resistance and increased susceptibility make hybrid breeding for reduced mycotoxin contamination a critical task. Despite the imperative for selecting maize hybrids with decreased susceptibility to fungal infection, the observed variation suggests that a significant array of maize-producing farmers choose hybrids less susceptible to fungal infection. This generally indicates the need for targeted educational initiatives, and disseminating knowledge about the benefits of selecting hybrids with documented resistance traits to enhance food safety and reduce mycotoxin and associated risks in maize production systems.

### 3.3. Interactions between Nitrogen Fertilization and Hybrids

The results reveal that the nitrogen application complex pattern of individual mycotoxins was influenced by changes resulting from the impact of the hybrid. While the interaction between nitrogen application rate and maize hybrids was evident in the changes in mycotoxin concentration in the kernels of the three maize hybrids, the dependence was different. There was an additive interaction in the trend in DON contamination with nitrogen fertilization rate, with a reversed impact following the hybrid’s responses. The tolerant hybrid DKC4590 showed less contamination compared to the susceptible hybrid GKT376. Interestingly, the medium nitrogen rate (N90) demonstrated relatively better results in 2022 than in 2023. However, there were no discernible differences between GKT376 and the hybrid P9610 with unknown tolerance features across all nitrogen doses, suggesting the impact of hybrid genetics irrespective of nitrogen dose. The effects of the FB accumulation interaction between nitrogen fertilization and hybrids were inconclusive. In 2022, significantly higher FB levels were observed at N150, while in 2023, significantly higher levels were recorded at the control (N0), with lower levels at the highest nitrogen rate (N150). This variability suggests that manipulating these two factors can yield similar or dissimilar mycotoxin levels in different maize hybrids, creating two environments with high and low mycotoxin risk. 

**Figure 2:**
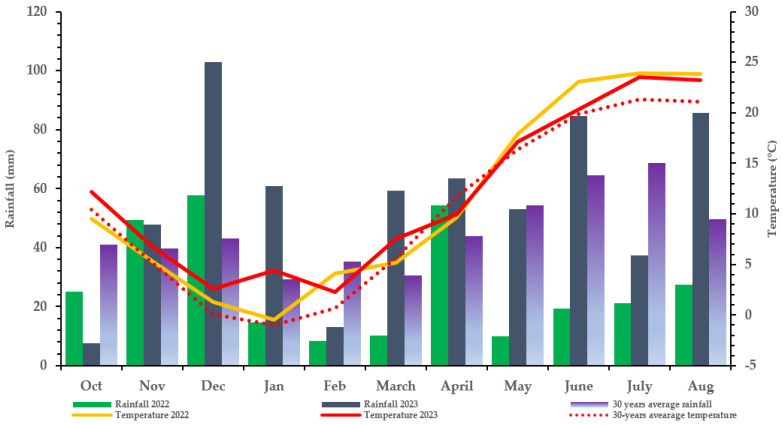
Meteorological data at the Látókép experimental station of the University of Debrecen (Debrecen: 2022, 2023, Hungary).

Despite the % average aspergillus ear rot being higher in 2023 (0.32%) than in 2022 (0.28%), only 2022 recorded AFB, which indicated a significantly higher AFB1 level (128.56 ppb) at N150 and the lowest level at N0. This stresses that the visual assessment of AF% (mere existence of fungal mycelium) does not reflect the amount of aflatoxin produced. This is further proved by *A. flavus*-inoculated treatment with an average mean ear rot of 0.05–0.28% in 2022, which resulted in an elevated AFB1 toxin for the hybrid than the AF% of 0.03–0.32% in non-inoculated and inoculated controls, with no AFB1 produced compared to 2022. This could be because mycotoxins arise from intricate metabolic processes brought on by the fungal adaptation to varying environmental and climatic conditions and stressors [45]. The conditions and stressors include the availability of nutrients, temperature, moisture content, water activity, relative humidity, substrate, and fungicide use, which are crucial in identifying the types of fungi that grow and influence crop mycotoxin production [46]. In this study, rainfall (moisture content) and humidity, in particular, could relatively influence the ear rot severity and mycotoxin contamination of *A. flavus* and *F. graminearum*, which vary significantly between the two study years (Figure 2).

Mycotoxins have a direct correlation with fungal infections [46]; this is in line with our findings, where each respective fungal severity positively correlated to the toxin produced (Figure 1); however, the results indicate the co-occurrence of aflatoxin and FB (Table 4), in line with Casu et al. [47]. This co-occurrence poses a further worry as the co-contamination of agricultural commodities results from the simultaneous infection of crops by mycotoxigenic fungal strains and the synthesis of multiple mycotoxins by the same strain, which could result from synergistic interactions. Therefore, the influence of environmental factors is crucial to further determining the additive interaction of various factors. 

## 4. Materials and Methods

### 4.1. Experimental Site and Treatments 

A field experiment was conducted in 2022 and 2023 at the Látókép long-term research site of the University of Debrecen, located at 47°33′42″ N; 21° 27′02″ E, Debrecen, Hungary. The soil conditions are homogenous calciferous Chernozem formed on the Hajdúság loess ridge, with an upper layer humus content average of 2.7–2.8% and a thickness of around 0.8 m. The acidity of the upper soil layers is almost neutral (pHKCl = 6.46–6.6). The phosphorus supply of the calcareous soil is average (AL-soluble P_2_O_5_ 133 mg kg^−1^), while its potassium supply is average–good (AL-soluble K_2_O 240 mg kg^−1^). The soil plasticity index (KA) was between 43 and 47.6. The experiment point contained sunflowers as the previous crop. The soil moisture content during sowing was suitable for germination in both years. However, during the 2022 season, May, June, and July were dry. Therefore, two instances of (2) supplementary irrigation were performed in May and early July during the critical fertilization, silking, and grain-filling periods. The meteorological data at the Látókép experimental station of the University of Debrecen for 2022 and 2023 are as presented in Figure 2.

The experiment was carried out in a quadruplicate using a split–split plot design. The reason for the design was that hybrids were assumed to be fixed effects and that the relationship between N and mycotoxin production was more of interest than a direct comparison between the effects of various levels of N fertilizer on mycotoxin contamination. The main plots were nitrogen rates (0, 90, and 150 KgNha^−1^), the sub-plot treatment was the three (3) commercial maize hybrids Table 5, and the sub-subplot treatment was the fungal species ear inoculation. The subplot size was 5 m × 3.04 m (15.2 m^2^) with a spacing of 76 cm between rows, making four rows of plots consisting of approximately 25 plants each and a distance of 1 m between blocks. Three rows were exposed to artificial inoculation; according to international guidelines, one inoculum was employed for a single pathogen. One with a strain of *F. graminearum*, one with a strain of *F. verticillioides* and the third raw sample was inoculated with a strain of *A. flavus*. The untreated check was the fourth raw. 

### 4.2. Isolates and Inoculation 

**Figure 3:**
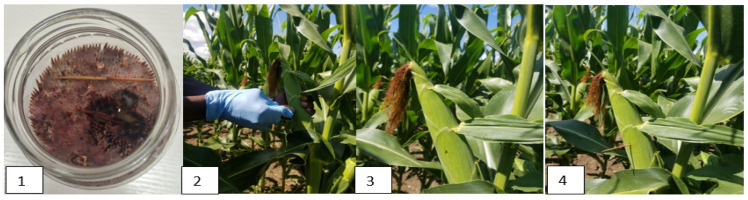
Illustrates the toothpick method inoculation process, depicting the sequence of steps: (**1**) infested toothpick, (**2**) creating an aperture, (**3**) punctured ear, (**4**) toothpick inserted.

All strains of *F. graminearum*, *F. verticillioides*, and *A. flavus* included in this trial were isolated in Hungary from naturally infected grain and were part of the microorganism collection of the Cereal Research Nonprofit Ltd. (Gabonakutató Nonprofit Kft: Szeged, Hungary) GK- Szeged, Hungary. Inocula preparation was performed according to the protocol of Szabó et al. [30]. Inoculation was conducted by toothpick [3] to evaluate kernel resistance (Figure 3). It was performed six days after 50% mid-silking by inserting infested toothpicks into the middle of the upper ear in a hole made by an awl, 15 mm long and 1.5 mm wide, and left until the harvesting period.

### 4.3. Evaluation of Ear and Kernel Rot Severity

To ensure improved sampling, the evaluation considered only the ears on which the toothpick’s mark was identified, using an average of 15 inoculated cobs per plot with each fungal isolate. At maturity, with an allowable harvesting moisture content of around 15–17%, the cobs were manually harvested and dehusked to evaluate the severity of the ear and kernel rot. The severity of fungus-induced ear rot was calculated using the percentage scale [3]. Based on this procedure, it was given as the percentage of ear coverage of an average regular ear (700–800 grains). It explains that a 1% infection is obtained when the ear contains 7–8 kernels with visible infection, and a 0.15% is obtained when only one kernel shows visible infection. The recording of severity involved two values, one directly from toothpick infection and the other independent infection as natural. 

### 4.4. Sample Preparations and Measurement of Mycotoxins 

For the toxin study, five ears with an average prevalence of ear rot without insect damage were selected per row. The ears were collected in a mesh-lined Rashel bag, stored under dry conditions until dry for three weeks, and then hand-shelled to avoid potential contamination. The grains were rough ground and carefully mixed, then finely milled for toxin measurement. The milling was carried out using a Perten laboratory mill (T: 3310, PI, 126 53 Hagersten, Sweden).

Mycotoxin contamination was measured using an enzyme-linked immunosorbent assay (ELISA) test. The amount of total fumonisin (FUM), DON, and AFB1 was measured by AgraQuant Fumonisin 0.25/5.0 ELISA kit, AgraQuant Deoxynivalenol 0.25/5.0 ELISA kit and AgraQuant Aflatoxin B1 2/50 ELISA kit, manufactured by Romer Labs, Tulln, Austria, respectively. According to the manufacturer’s instructions, the assays were carried out on ground maize kernel samples using a direct competitive assay. The samples were measured at 450 nm using a microtiter plate reader (Thermo Scientific, Waltham, MA, USA). Measurements were repeated four times with CV% < 15%. The ELISA kit detection limits were 0.2 ppm for DON and FB and 2 ppb for AFB1, respectively.

### 4.5. Statistical Data Analysis 

The influence of N rates and maize hybrids on the severity of ear and kernel coverage and mycotoxin contamination levels was determined by variance analysis after subjecting the data to a normality test using the Shapiro–Wilk test. The treatment means were compared using LSD and considered significant when *p* < 0.05. The statistical software Genistat 18th edition, version 18.2 (64 bit) registered for Plant Research International, was used. Additionally, the built-in Excel function (analysis toolpack) was used to perform Pearson correlation analysis (two-tailed) and regression analysis, investigating the relationships between the severity of toxin production within and between toxigenic fungal species.

## Figures and Tables

**Table 1:** Effects of nitrogen fertilization, hybrids and inoculation treatments on ear rot severity and mycotoxin contamination (Debrecen, 2022 and 2023).

Source of Variation		Ear Rot Severity (%)			Mycotoxin Contamination		
		AF	FV	FG	AFB1 (ppb)	FB (ppm)	DON (ppm)
**Nitrogen fertilization rates**							
**2022**	**Ø**	0.08 ^a^	0.07 ^a^	7.6 ^a^	9.83 ^a^	1.17 ^a^	4.37 ^a^
	**N90**	0.21 ^b^	0.16 ^b^	13.1 ^a^	43.11 ^ab^	2.39 ^b^	3.84 ^a^
	**N150**	0.22 ^b^	0.26 ^c^	13.7 ^a^	67.9 ^b^	2.89 ^b^	3.96 ^a^
**2023**	**Ø**	0.15 **^ab^**	0.17 **^b^**	23.5 **^b^**	<LOD	2.69 **^ab^**	60.3 **^b^**
	**N90**	0.21 **^b^**	0.24 **^c^**	24.9 **^b^**	<LOD	3.57 **^b^**	59 **^b^**
	**N150**	0.18 **^ab^**	0.16 **^b^**	25.1 **^b^**	<LOD	1.88 **^a^**	50.5 **^b^**
	***LSD*** **_(0.05)_**	**0.1222**	**0.0864**	**7.305**	**34.265**	**1.048**	**26.960**
**Maize hybrid effects**							
**2022**	**DKC4590**	0.09 ^a^	0.13 ^a^	7.3 ^a^	37.08 ^a^	1.73 ^a^	2.11 ^a^
	**GKT376**	0.22 ^b^	0.24 ^c^	9.6 ^a^	51.38 ^a^	2.62 ^a^	4.73 ^a^
	**P9610**	0.19 ^ab^	0.11 ^a^	17.5 ^b^	32.38 ^a^	2.11 ^a^	5.34 ^ab^
**2023**	**DKC4590**	0.21 **^ab^**	0.21 **^bc^**	15.8 **^b^**	<LOD	2.51 **^ab^**	37.1 **^bc^**
	**GKT376**	0.17 **^ab^**	0.22 **^bc^**	27.7 ^c^	<LOD	3.7 **^b^**	66.5 **^c^**
	**P9610**	0.16 **^ab^**	0.14 **^ab^**	29.9 **^c^**	<LOD	1.94 **^a^**	66.2 **^c^**
	***LSD*** **_(0.05)_**	**0.1205**	**0.0916**	**6.740**	**44.364**	**0.983**	**32.269**
**Inoculation effects**							
**2022**	**Treated**	0.282 ^b^	0.325 ^b^	22.9 ^b^	62.59 ^b^	3.02 ^b^	8.11 ^a^
	**Untreated control**	0.053 ^a^	0.001 ^a^	0.1 ^a^	17.97 ^a^	1.29 ^a^	0.34 ^a^
**2023**	**Treated**	0.321 **^b^**	0.327 **^b^**	48.8 ^c^	<LOD	4.66 **^c^**	113.2 **^b^**
	**Untreated control**	0.034 **^a^**	0.052 **^a^**	0.1 ^a^	<LOD	0.77 **^a^**	<LOD
	***LSD*** **_(0.05)_**	**0.0887**	**0.0838**	**5.519**	**28.877**	**0.945**	**25.002**

Letters in the column are significant (*p* < 0.05); LOD—Limit of detection; AF = *A. flavus*; FV = *F. verticillioides*; FG = *F. graminearum;* AFB1 = aflatoxin B1; FB = fumonisin B1 + B2; DON = deoxynivalenol.

**Table 2:** Interactive effects of nitrogen fertilizer application rate and hybrid genotypes on-ear and kernel rot severity (Debrecen, 2022, 2023).

	N Level	Hybrids	Artificial Inoculated			Mean	Untreated Control		Mean
			AF%	FV%	FG%		Aspergillus (%)	Fusarium (%)	
**2022**	**Ø**	**DKC4590**	0.04	0.09	10.30	3.48	0.01	0.10	0.06
		**GKT376**	0.15	0.22	9.60	3.32	0.00	0.00	0.00
		**P9610**	0.21	0.12	25.60	8.64	0.05	0.00	0.03
	**N90**	**DKC4590**	0.29	0.14	19.70	6.71	0.08	0.10	0.09
		**GKT376**	0.34	0.57	19.50	6.80	0.09	0.11	0.10
		**P9610**	0.24	0.21	39.00	13.15	0.21	0.10	0.16
	**N150**	**DKC4590**	0.07	0.57	13.90	4.85	0.03	0.00	0.02
		**GKT376**	0.77	0.66	28.20	9.88	0.01	0.10	0.06
		**P9610**	0.43	0.34	40.10	13.62	0.01	0.20	0.11
	**Mean**		**0.28**	**0.32**	**22.88**	**7.83**	**0.05**	**0.08**	**0.07**
**2023**	**Ø**	**DKC4590**	0.39	0.37	34.20	11.66	0.10	0.13	0.11
		**GKT376**	0.20	0.25	49.90	16.78	0.00	0.10	0.05
		**P9610**	0.23	0.34	55.90	18.82	0.00	0.15	0.08
	**N90**	**DKC4590**	0.29	0.29	22.90	7.83	0.20	0.15	0.18
		**GKT376**	0.40	0.56	59.00	19.99	0.00	0.00	0.00
		**P9610**	0.39	0.27	67.30	22.65	0.00	0.00	0.00
	**N150**	**DKC4590**	0.26	0.25	37.10	12.54	0.00	0.05	0.03
		**GKT376**	0.40	0.38	56.90	19.23	0.00	0.15	0.08
		**P9610**	0.34	0.23	55.90	18.82	0.00	0.05	0.03
	**Mean**		**0.32**	**0.33**	**48.78**	**16.48**	**0.03**	**0.09**	**0.06**
	***LSD*** **_(0.05)_**		**0.2045**	**0.1544**	**11.517**		**0.1722**		

AF% = percentage of kernels damaged by *A. flavus*; FV% = percentage of kernels damaged by *F. verticillioides*; FG% = percentage of kernels damaged by *F. graminearum*.

**Table 3:** Interactive effects of nitrogen fertilizer application rate and hybrid genotypes on the amount of mycotoxin contamination (Debrecen, 2022, 2023).

	N Level	Hybrids	Artificial Inoculated			Untreated Control		
			DON (ppm)	FB (ppm)	AFB1 (ppb)	DON (ppm)	FB (ppm)	AFB1 (ppb)
2022	Ø	DKC4590	4.11	0.97	19.95	<LOD	0.47	4.44
		GKT376	9.78	2.49	15.94	<LOD	0.93	4.23
		P9610	12.33	1.71	14.41	<LOD	0.48	<LOD
	N90	DKC4590	5.82	3.06	26.68	<LOD	2.34	14.21
		GKT376	8.98	3.90	124.2	<LOD	1.48	0.88
		P9610	8.21	2.26	29.99	<LOD	1.33	62.67
	N150	DKC4590	2.71	2.37	119.4	<LOD	1.15	37.8
		GKT376	9.60	6.59	128.56	3.10	0.33	34.46
		P9610	11.48	3.81	84.2	<LOD	3.09	3.00
	Mean		8.11	3.02	62.59	3.1	1.29	20.21
2023	Ø	DKC4590	54.40	5.34	<LOD	<LOD	1.09	<LOD
		GKT376	154.60	5.19	<LOD	<LOD	0.66	<LOD
		P9610	152.90	3.87	<LOD	<LOD	<LOD	<LOD
	N90	DKC4590	91.40	4.33	< LOD	<LOD	1.99	<LOD
		GKT376	120.90	8.81	<LOD	<LOD	1.44	<LOD
		P9610	141.50	4.86	<LOD	<LOD	<LOD	<LOD
	N150	DKC4590	76.60	2.32	<LOD	<LOD	<LOD	<LOD
		GKT376	123.90	4.60	<LOD	<LOD	1.47	<LOD
		P9610	102.40	2.59	<LOD	<LOD	0.3	<LOD
	Mean		113.17	4.66			1.16	
*LSD*_(0.05)_			52.347	1.717	70.469		1.717	70.469

AFB1 = aflatoxin B1; FB = fumonisin B1 + B2; DON = deoxynivalenol; LOD—Limit of detection.

**Table 4:** Coefficient values of Pearson correlation showing a relationship between fungal species ear rot severity and mycotoxin contamination (Debrecen, 2022, 2023).

	AF%	FV%	FG%	DON (ppm)	FB (ppm)	AFB1 (ppb)
AF%	1					
FV%	0.57917	1				
FG%	0.53016	0.17876	1			
DON (ppm)	0.15678	−0.0168	0.60933	1		
FUM (ppm)	0.69222	0.73135	0.24318	0.2631	1	
AFB1 (ppm)	0.22637	0.44445	−0.0351	−0.3457	0.15046	1

AF% = percentage of kernels damaged by *A. flavus*; FV% = percentage of kernels damaged by *F. verticillioides*; FG% = percentage of kernels damaged by *F. graminearum*; AFB1 = aflatoxin B1; FB = fumonisin B1 + B2; DON = deoxynivalenol. Green color = positive Correlation, Yellow color = No correlation, Red Color = negative correlation.

**Table 5:** Characteristics of selected maize hybrids.

Company and Hybrid Code	Type	Characters
Pioneer (P9610)	Commercial	Unknown sensitivity and High-yielding
Bayer (DKC4590)	Commercial	DON, FUM and AFB tolerant
Cereal Research Nonprofit Ltd-GK Szeged (GKT376)	Commercial	DON, FUM and AFB susceptible

## Data Availability

The data for the findings of the current study are available from the corresponding author upon request.
